# Looking back to look forward: Surviving COVID-19 and the future of the NHS

**DOI:** 10.1177/14782715231173667

**Published:** 2023-05-12

**Authors:** Adam Vacek, Siddharthan Chandran, Linda Bauld, Carey Lunan, Andrew Horne, Steve Kerr, Caroline Whitworth, Wojcik Wojtek

**Affiliations:** 1 Edinburgh Medical School, University of Edinburgh, Edinburgh, UK; 2 Usher Institute, Edinburgh Medical School, University of Edinburgh, Edinburgh, UK; 3 GP partner Craigmillar Medical Group, past-Chair of RCGP, Edinburgh, UK; 4 MRC Centre for Reproductive Health, University of Edinburgh, Edinburgh, UK; 5 Patient representative on NHS Lothian Healthcare Governance Committee, Edinburgh, UK; 6 Medical Director Acute Services, NHS Lothian, Edinburgh, UK; 7 Department of Psychological Medicine, Royal Infirmary of Edinburgh, Edinburgh, UK

**Keywords:** NHS, COVID-19, future, expectations, healthcare, debate

## Abstract

The COVID-19 pandemic affected healthcare systems worldwide, including the National Health Service (NHS). It drastically changed the practice and delivery of healthcare and laid bare longstanding structural flaws. It also brought a time of innovation and digitalisation and renewed appreciation of the role of public health. This paper offers a thematic summary of a debate held in December 2021 by the University of Edinburgh School of Medicine. It featured a multi-specialty panel of doctors and patient representative discussing the likely impact of the pandemic on the future of NHS. It serves as a reflection point on the pressures the NHS has faced since and their likely genesis at a time when the impact of the pandemic on staff risks being forgotten.

## Introduction

The COVID-19 pandemic affected the practice and delivery of healthcare, including the training of healthcare staff. Depending on one’s perspective, it has been a catalyst for innovation and transformation, while also laying bare longstanding structural challenges in healthcare, resulting in even greater health inequality across society.

As the UK’s National Health Service (NHS) enters a new period of difficulty and pressure around waiting times, staffing and resourcing in the context of inflationary pressures and the cost-of-living crisis, this paper revisits a debate held by the University of Edinburgh Medical School in December 2021 at a time when pandemic restrictions were easing, prompting reflection on what its legacy on the NHS would be.

The panel included representation from – public health policy, general practice, women’s health, patient experience, medical students, academic and hospital medicine. The themes remind us of the trajectory the UK NHS has been on; and that it sometimes pays to look back in order to look forward.

## Managing expectations in primary care

At the beginning of the pandemic, the NHS experienced an outpouring of gratitude from the public. As the pandemic progressed, people understandably became more anxious about the lack of ‘return to normal’, or what the ‘new normal’ would look like.

From a general practice perspective, the pandemic exposed the existing gaps in social and health inequalities that already existed in Scotland, disproportionately affecting the least well-off and certain ethnic minority groups.^1,2^ As the efforts to recover and redesign the NHS continue, discussions take place about how we do that to ensure a more equitable system.

General practice has suffered the challenge of negative public perceptions, despite adapting models of consulting during the pandemic to minimise infection risk and include telephone and video-based consultations where face-to-face consultations were not clinically necessary. Despite an initial dip in consulting numbers at the start of the pandemic, these numbers then began to rise rapidly. Many practices now report higher workloads than they did pre-pandemic.^3^ It is important to understand what is driving these negative perceptions, as they are damaging to professional morale and public trust. Are they driven by people’s day-to-day experience of trying to get through on busy phonelines? Or having to wait for a routine appointment? Or not been automatically offered in-person appointments? Additionally, social services have shouldered challenges during the pandemic, which ultimately contribute to longer waits on packages of care and placements, further compounding the frustration of patients and families.^4^

In the face of staffing shortages in primary care, an increasing, and ageing population, and rising demand and workload, perhaps a ‘national conversation’ is now needed about the future of the NHS? In many ways, the NHS is a victim of its own success, as the world has changed immeasurably since its inception in 1948. Rather than viewing the NHS as failing, do we instead need to find a way to de-politicise the NHS and consult with the public about what matters to them? What are the priority areas, what can the NHS continue to reasonably provide, and what can it not, given its current funding levels? Are its founding principles still valued? For the Scottish Government to deliver on strategies such as Realistic Medicine, these difficult conversations need to be a part of that.

## Funding

People in Scotland and across the UK are fortunate to have one of the most equitable and accessible healthcare of almost any country in the world. About 15 billion pounds (22% of the government’s public spending) is spent each year on the NHS, employing over 150 thousand people, and many more in other organisations linked to the NHS.^5^ Is the NHS adequately funded, given the scale of the challenge it is facing?

During 2020/2021, there was a 50% reduction in inpatient procedures and a 41% reduction in outpatient activity, and now the NHS is trying to catch up.^6^ The Scottish Government has committed to around 20% increase in activity in its recovery plan over the next 5 years.^7^ Whether that will be enough to address the backlog is hard to judge. Almost a year since the debate, we can see that the backlog numbers remain high.^6^ Governments must balance total resources available – while the NHS has many financial needs and requires more money, that also means there will be less to spend elsewhere. This becomes even more pertinent with the developing economic situation in the UK and multiple requests from other public services for increased funding.

## Privatisation of care

As of December 2021, the system was delivering about 50% of elective surgery compared to pre-COVID-19, but with an increase in referrals over the same period.^6^ Some patients inevitably turned to private healthcare because of prolonged waiting times.

Some argue for more integrated ways to work with the private sector, as the NHS waiting times are simply getting longer and longer, which is having negative knock-on effects. Another view is of concern for a drift towards a two-tier healthcare system, whereby private providers offer profitable interventions to healthier, wealthier people. This may increase health inequalities and contribute to the historical concern (first raised by Nye Bevan) of the NHS being allowed to fail gently by stealth, rather than accountably by an explicit policy decision.

In an increasingly difficult economic situation, the question was posed of how to achieve a longer-term investment strategy, to aid the recovery of performance in the NHS.

## Impact on workforce and staff morale

The greatest asset of the NHS is the commitment and goodwill of its staff. As staff continued to go above and beyond, and there was a concern that it would become a normalised expectation long-term, impacting on retirements, recruitment and retention.^8,9^ What early in the pandemic was a willingness to prioritise NHS work over private life decayed over time, with staff reporting moral distress within the NHS due to the challenge of delivering good patient care in the face of staff shortages, individual mental fatigue or lack of time for emotional support.^8^

## Impact on training and education

As for medical education, the changes made during and after the pandemic will have long-lasting impacts on training medical students and junior doctors. Recent movements surrounding the devaluing salary of junior doctors and the working and training conditions in the NHS are some of the issues the COVID-19 pandemic brought to light.^10^

The pandemic backlog and the increased adoption of digital technology could provide medical students and junior doctors with both costs and opportunities. It may facilitate increased clinical contacts. However, with the increased workload in the NHS, dedicated protected time for teaching and training risks are being eroded.^10,11^ Many junior doctors kept pushing forward very hard and remained resilient during the pandemic, serving as great role models for medical students. However, they have also had to deal with emotionally devastating situations as a result of very sick patients, at times without appropriate support from senior colleagues and in poor working conditions. This may have a significant impact on the retention of junior doctors: the term ‘Drexit’ (Doctor-Exit) has been coined to describe the increasing number of doctors leaving the NHS for overseas work.^12^

Additionally, improvements in equality, diversity and inclusion training for healthcare workers and students may have improved the understanding of how marginalised groups were impacted at the start of the pandemic. The emotional labour of jobs in the NHS is easily underestimated^13^ with what has been described as a lack of support for the ‘psychological masonry’ that flies around in the everyday work of healthcare professionals.^13^ The point was made that more should be done to help maintain compassionate practice and the well-being of staff. It remains relevant today.

## Public health and healthcare politics during the pandemic

There has variously been a lot of blame directed at politicians for the handling of the pandemic. Few politicians are health professionals or have a background in public health. Governments across the UK and in many other countries drew on advice from clinical and public health advisers and advisory groups in developing pandemic policies. In Scotland, the Chief Medical Officer formed a COVID-19 advisory group and relevant subgroups. Some decisions made in circumstances of significant uncertainty can be viewed differently with the benefit of hindsight. Some have argued that the UK adopted an approach that did not sufficiently take into account or learn from the experiences of other countries which experienced Ebola and SARS outbreaks, for example. Public enquiries are now underway as well as analysis of the response in a range of countries.^14^

This experience may, however, have modelled for students and younger doctors the opportunities to become involved in health policy work and effect change. An example of this would be the creation of the Homeless and Inclusion Health Society by students of Edinburgh Medical School.^15^ At the University of Edinburgh, medical students have been very involved with their feedback about changes to the curriculum post-pandemic, ensuring high-value education for their future colleagues. This resulted in substantial improvements to clinical medicine teaching in Edinburgh.

## Equality and diversity in healthcare

The pandemic highlighted fundamental structural inequalities in housing, resources and jobs, all of which ultimately affect each other and people’s health. This reinvigorated debates about the intersectional nature of inequality and about the need for better integration of the NHS with social and other services to improve health outcomes. Personalised plans could be developed better recognising that people have different needs and circumstances and how to find effective and sensitive ways to address those. Arguably, the increased diversity of the medical workforce, no longer a profession of White, middle-class men, may help. Widening participation in medical school is one of the ways that we can ensure a demographic that better represents the populations that it cares for.

## Digitalisation of healthcare

COVID-19 served as a catalyst for innovation and digitalisation. While this brings benefits, it brings a risk of digital exclusion. For example, at the beginning of the pandemic, GP practices were encouraged to move to video conferencing instead of in-person consulting, although many found the technology initially problematic.^3^ Subsequently, there were many more telephone consultations, backed up with clinical photographs if needed.^3^ In order to participate, patients need reliable access to smartphones, internet connectivity, good signal and private spaces. It can be additionally difficult if they do not speak English as a first language.

While digital appointments may be cheaper and more efficient in terms of travel and time off work, the technology required risks excluding some people. It may not capture as much clinical information as a face-to-face appointment and observing a patient walking in. The question was posed of the evaluation needed into the impact of more digital appointments on appointment times, the number of investigations and referrals requested and longer-term health outcomes. Digital healthcare is here to stay and can work well for certain types of health problems. A hybrid model is likely but is there a risk that greater use of digital healthcare could significantly worsen existing health inequalities?

## Healthcare advocacy and education

When the NHS is under strain, it may drive more patients to adopt self-management approaches. Individual healthcare advocacy can be a valuable tool to help people remain healthy and manage their conditions well. However, while empowering people to look after their health is good, this is not equally easy for everyone. Some people are in a better position to be able to manage their health than others – health education, functional and health literacy, economic advantage and ease of access to quality information also play a role here. While enabling individuals to self-care when this is appropriate was viewed as positive, it was also recognised that some avoided seeking healthcare, either in the community or at a hospital, sometimes because of fear, confusion or guilt. For many, who did need to consult with a clinician, this led to worse outcomes.

The pandemic certainly brought some improvements in digital health education that have been viewed as positive.^16^ However, some preventable risk factors increased. For instance, there was a 20% increase in alcohol-attributable mortality during the pandemic, a worsening of the drug-related deaths crisis and a rise in obesity.^17,18^ Scotland is disproportionately affected by some of these issues. Preventative approaches are highly cost-effective, but they are one of the first services to be cut in response to economic pressures. Will the post-pandemic recognition of the importance of public health interventions assist their funding?

Health advocacy on social media expanded rapidly, competing with an increase in misinformation about coronavirus and vaccine safety. As more people turn to online resources for health-related information, it raises the question of how medicine could engage positively with social media and help the public understanding of how to access reliable health information online and not fall prey to misinformation, especially in some vulnerable groups.^19,20^

## Conclusion

The COVID-19 pandemic brought a period of enormous potential to make significant changes that could improve the healthcare system for both patients and staff. It also left the NHS at risk, facing both backlogs of patients waiting for treatment and staff shortages at a time of inflationary pressures and a cost-of-living crisis. The themes articulated above serve to remind us of how looking back may helpfully inform our understanding of the genesis of the pressures it currently faces.
